# Nesfatin-1 in the Lateral Parabrachial Nucleus Inhibits Food Intake, Modulates Excitability of Glucosensing Neurons, and Enhances UCP1 Expression in Brown Adipose Tissue

**DOI:** 10.3389/fphys.2017.00235

**Published:** 2017-04-24

**Authors:** Jun-hua Yuan, Xi Chen, Jing Dong, Di Zhang, Kun Song, Yue Zhang, Guang-bo Wu, Xi-hao Hu, Zheng-yao Jiang, Peng Chen

**Affiliations:** ^1^Special Medicine Department, Medical College, Qingdao UniversityQingdao, China; ^2^Physiology Department, Medical College, Qingdao UniversityQingdao, China; ^3^Clinical Medicine Department, Medical College, Qingdao UniversityQingdao, China; ^4^Department of Human Anatomy, Histology and Embryology, Qingdao UniversityQingdao, China

**Keywords:** food intake, energy expenditure, glucose sensitive neurons, lateral parabrachial nucleus, nesfatin-1, SHU9119, rat

## Abstract

Nesfatin-1, an 82-amino acid neuropeptide, has been shown to induce anorexia and energy expenditure. Food intake is decreased in *ad libitum*-fed rats following injections of nesfatin-1 into the lateral, third, or fourth ventricles of the brain. Although the lateral parabrachial nucleus (LPBN) is a key regulator of feeding behavior and thermogenesis, the role of nesfatin-1 in this structure has not yet been delineated. We found that intra-LPBN microinjections of nesfatin-1 significantly reduced nocturnal cumulative food intake and average meal sizes without affecting meal numbers in rats. Because glucose sensitive neurons are involved in glucoprivic feeding and glucose homeostasis, we examined the effect of nesfatin-1 on the excitability of LPBN glucosensing neurons. *In vivo* electrophysiological recordings from LPBN glucose sensitive neurons showed that nesfatin-1 (1.5 × 10^−8^ M) excited most of the glucose-inhibited neurons. Chronic administration of nesfatin-1 into the LPBN of rats reduced body weight gain and enhanced the expression of uncoupling protein 1 (UCP1) in brown adipose tissue (BAT) over a 10-day period. Furthermore, the effects of nesfatin-1 on food intake, body weight, and BAT were attenuated by treatment with the melanocortin antagonist SHU9119. These results demonstrate that nesfatin-1 in LPBN inhibited food intake, modulated excitability of glucosensing neurons and enhanced UCP1 expression in BAT via the melanocortin system.

## Introduction

Nesfatin-1 was identified as a satiety molecule by Oh et al. (2006), and its precursor, nucleobindin-2 (NUCB2), is associated with severe obesity in humans (Zegers et al., 2012). NUCB2/nesfatin-1 is widely expressed in the periphery (García et al., 2012) and in the central nervous system. It is expressed mainly in the paraventricular nucleus of hypothalamus (PVN), arcuate nucleus, lateral hypothalamic area, solitary tract nucleus, and the spinal cord (Oh et al., 2006; Brailoiu et al., 2007; Maejima et al., 2009). Additionally, expression of NUCB2/nesfatin-1 has been detected in the lateral parabrachial nucleus (LPBN) (Goebel et al., 2009, 2011a).

The LPBN is involved in appetite regulation (Wu et al., 2012; Garfield et al., 2015; Roman et al., 2016), thermostasis (Morrison et al., 2012), glucose homeostasis (Flak et al., 2014), and malaise (Carter et al., 2015). Various feeding-related peptides, including leptin (Alhadeff et al., 2014a), glucagon-like peptide-1 (GLP-1) (Alhadeff et al., 2014b; Richard et al., 2014), amylin (Lutz, 2010), and cholecystokinin (Becskei et al., 2007), act directly on the LPBN to reduce food intake. However, evidence for the role of nesfatin-1 in the LPBN has hitherto been lacking. Acute injections of nesfatin-1 into the lateral (Maejima et al., 2009), third (Oh et al., 2006; Watts and Donovan, 2010), or fourth (Yosten and Samson, 2009) ventricles of the brain decrease food intake in rats and mice. The anorexigenic mechanisms of nesfatin-1 encompass several hypothalamic and medullary pathways in a leptin-independent manner (Oh et al., 2006; Maejima et al., 2009; Stengel et al., 2009a). The crosstalk between nesfatin-1 and corticotropin-releasing factor, oxytocin, and melanocortin pathways has yet to be clarified.

Glucose sensitive neurons are involved in glucoprivic feeding and glucose homeostasis. Recently they have received more attention due to their potential role in regulating appetite (Routh, 2002; Watts and Donovan, 2010). Previously, we discovered that nesfatin-1 inhibits food intake by influencing the firing rates of glucose sensitive neurons in the hypothalamus (Chen et al., 2012) and the dorsal vagal complex (Dong et al., 2014). It was also shown recently that the glucose-sensitive territory in the brain includes the LPBN in addition to the hypothalamic and hindbrain centers (de Araujo, 2014; Garfield et al., 2014). Moreover, GLP-1 decreases food intake and increases the firing rates of LPBN neurons (Richard et al., 2014). However, the types of neurons responding to GLP-1 have not been identified. Here, we investigate the influence of nesfatin-1 in the LPBN on the firing rate of glucosensing neurons in this area and its effect on feeding behaviors.

Central administration of nesfatin-1 has been shown to increase energy expenditure in rodents (Konczol et al., 2012; Wernecke et al., 2014). In addition, its anorexigenic effects are abolished by a melanocortin 3/4 receptor antagonist, SHU9119 (Oh et al., 2006). In fact, the central melanocortin system and its interactions with other neuroendocrine pathways have emerged as key regulators of appetite and thermogenesis (Myers and Olson, 2012; Krashes et al., 2016). A mutation in the melanocortin-4 receptor has been associated with severe obesity and inducing lower energy expenditure in humans (Ma et al., 2004). Administration of the melanocortin 3/4 receptor agonist MTII into the LPBN of rodents significantly reduces nocturnal food intake and increases core temperature and heart rate without affecting spontaneous activity (Skibicka and Grill, 2009). Intriguingly, Williams et al. (2003) found that the forebrain and hindbrain were equally effective at stimulating MTII-induced expression of uncoupling protein 1 (UCP1), a primary effector of thermogenesis, in brown adipose tissue (BAT). We hypothesize that the LPBN melanocortin-signaling pathway is involved in the effects of nesfatin-1 on food intake, energy expenditure, and body weight. We tested this hypothesis by determining whether the effects of nesfatin-1 on energy balance are blocked by SHU9119.

These studies utilized a combination of behavioral, electrophysiological, and pharmacological techniques to test the hypothesis that nesfatin-1 in the LPBN is involved in energy metabolism.

## Materials and methods

### Animals

Adult male Wistar rats (Qingdao Institute of Drug Control) weighing 270–300 g were housed in a temperature-controlled (23 ± 2°C) animal room (illumination from 7:00 to 19:00). Rats were group-housed (four per cage) and allowed free access to standard food and tap water for at least 1 week to adapt to their surroundings. The protocols were approved by the Qingdao University Animal Care and Use Committee in accordance with the National Institutes of Health guidelines.

### Surgery

Rats were anesthetized with chloral hydrate (80 mg/ml, 0.5 ml/100 g of body weight, i.p.) and positioned in a stereotaxic apparatus (SN-3; Narishige, Tokyo, Japan) for implantation of a 26-gauge chronic guide cannula above the right lateral LPBN. Stereotaxic coordinates were obtained from the brain atlas of Paxinos and Watson (Gaxinos and Watson, 2007): 9.0 mm caudal to bregma, 2.2 mm right lateral, and 6.4 mm ventral to the skull (Supplementary Figures 1A,B). After quickly and accurately reaching the target depth, microabsorbent cotton was used for local hemostasis. The cannula was fixed in place with dental cement and a stainless-steel screw. A 28-gauge obturator (the same length as the cannula) was placed in the cannula.

After completion of all experiments, the rats were sacrificed and their brains were removed. Cannula placement was verified by injections of 0.5 μl pontamine sky blue, and the brains were fixed in a 4% formaldehyde solution and cut on a freezing microtome (Kryostat 1,720; Leica, Germany). Data from rats with injection sites in the correct location were included in the analyses (Supplementary Figure 2A).

### Experimental procedures

#### Experiment 1: effects of nesfatin-1 in the lpbn on nocturnal food intake and meal patterns

After the surgery, rats (*n* = 32) were housed individually for recovery for 5–7 days before being placed in metabolic cages (Feeding and Activity Analyser 47552-002; Ugo Basile, Italy) and habituated to powdered standard chow (Qingdao Daren Fortune Animal Technology) which was crashed by the crusher (Ronghao, RH-600A) and tap water (Ulman et al., 2008). The metabolic cages were monitored with load cells incorporating data acquisition software (51,800, Feed-Drink Monitoring System Ver. 1.31; Ugo Basile, Italy). Load cells sense the load of food and liquid every 5 s, thus recording their consumption, and monitor the frequency of food/liquid uptake by detecting stable or unstable weights. The crumbs produced by rats were collected in the front compartment for a precise evaluation of food consumption. Animals were administered drugs with a 28-gauge injector via a microsyringe extended 0.8 mm below the guide cannula. The drugs were delivered to the nuclei parenchyma at a speed of 0.25 μl/min. After the injection, the injector was kept in the cannula for another 5 min (Dong et al., 2014). Prior to testing, rats received saline injections (0.5 μl of 0.9% NaCl) through the guide cannula for 3 days as training for drug injections. On the experimental day, food was withdrawn from the metabolic cages at 15:00. Rats received injections (0.5 μl) of nesfatin-1 (50 pmol, 1–82; Phoenix Pharmaceuticals, Burlingame, CA, USA) or vehicle through the cannulae before the onset of the dark cycle. The protocol of microinjection and the doses selected were as described in our previous studies (Chen et al., 2012, 2015; Dong et al., 2014). Drug injections were administered at least 72 h apart.

Food was returned to the rats between 19:00 and 7:00 for continuous monitoring of meal patterns and water consumption, calculated as g/300 g body weight (Stengel et al., 2009a). The minimum meal amount was 0.5 g, and a new meal was considered based on an intermeal interval of 5 min.

#### Experiment 2: effects of nesfatin-1 in the LPBN on long-term body weight gain

After completion of experiment 1, rats (*n* = 24) were individually housed and received 10 daily LPBN microinjections of nesfatin-1 or vehicle. The dose of nesfatin-1/vehicle was the same as in experiment 1. Body weights were recorded manually immediately before injections on day 1 and 24 h after each injection.

#### Experiment 3: electrophysiological effects of nesfatin-1 on glucose-reactive neurons in the LPBN

Rats (*n* = 40) were anesthetized with urethane (1.0 g/kg body weight, i.p.) and placed in a stereotaxic apparatus. Supplemental anesthetics were added when necessary throughout the procedure. A rectangular portion of bone was removed to expose the brain, and LPBN nuclei were localized. The exposed dura mater was carefully cleaned, and one side of the sinus was ligated and cut to give way for the microelectrode. The exposed area was covered with warm agar (3–4% in saline) to improve the stability for recording, and the rat was covered by a cotton pad to maintain body temperature during the surgery. The barrels of a four-barrel glass microelectrode (3–10 μm) (Chen et al., 2012; Dong et al., 2014) were filled with the following solutions: 0.5 M sodium acetate in 2% pontamine sky blue for recording, 5 mM glucose solution, 0.9% NaCl, and 1.5 × 10^−8^ M nesfatin-1 solution (Chen et al., 2012, 2015). The latter three microelectrodes were connected to a four-channel pressure injector (PM2000B; Micro Data Instrument, Inc., USA) to eject drugs by gas pressure (Chen et al., 2012; Dong et al., 2014).

LPBN neurons were localized at 8.8–9.0 mm caudal to bregma, 2.0–2.4 mm from midline, and a depth of 6.5–6.8 mm from the skull (Gaxinos and Watson, 2007). Glucose sensitive neurons in the LPBN were identified by their activity in response to the 5 mM glucose solution. After recovery, 0.9% NaCl was given as a control. At least 3 min were allotted to ensure that the baseline firing was stable, and 120 s of baseline data were collected before drug application. Next, the nesfatin-1 solution was applied and the alterations in firing rates were recorded. The drug was considered to exhibit significantly excitatory or inhibitory effects based on an increase or decrease in the firing rate, respectively, of at least 20%. A drug effect was calculated when the maximal alteration of frequency appeared within 50 s after drug administration. Only data from rats with injection sites in the correct location were included in the analyses (Supplementary Figure 2B).

#### Experiment 4: influence of nesfatin-1 in the melanocortin system on energy balance

##### Effects of nesfatin-1 on feeding with SHU9119

Rats weighing 270–300 g underwent surgery as described in experiment 1 for the placement of unilateral guide cannulae and were divided into four groups (*n* = 12–14/group). Rats were individually housed and received daily injections (0.5 μl) through the cannulae into the LPBN for 10 days with 0.9% NaCl, nesfatin-1 (50 pmol), SHU9119 (250 pmol), or nesfatin-1 (50 pmol) + SHU9119 (250 pmol). Nesfatin-1 and SHU9119 (Sigma) were dissolved in 0.9% NaCl. Rats were weighed daily and food weights were recorded manually every hour for the first 6 h after injections and every 12 h thereafter (Stengel et al., 2009a). The doses of drugs were chosen based on the results of previous studies (Xu et al., 2015). The injection procedure was as described in experiment 1.

##### Adipose weight and morphometric analyse

Rats were sacrificed following the 10-day injection protocol. BAT from the interscapular region and unilateral white adipose tissue (WAT) from epididymal, inguinal, and perirenal regions were weighed. Portions of BAT were fixed in 4% formaldehyde in PBS (pH 7.4) for 24 h and embedded in paraffin before sectioning. Sections 10-μm thick were cut, were stained with Mayer's hematoxylin and eosin, and were imaged. Cell numbers were counted using Image J software (Unit area: 90 × 90 μm).

##### Western blot analysis

Remaining portions of BAT and WAT were frozen in liquid nitrogen and stored at −80°C until they were assayed for UCP1 protein levels. Adipose lysate proteins were subjected to 12% SDS-PAGE and electrotransferred to a polyvinylidene fluoride membrane (Millipore Corp., Billerica, MA, USA) for 2 h. After blocking, membranes were incubated overnight at 4°C in anti-UCP1 (ab10983; Abcam) and anti-beta-tubulin (2128S; Cell Signaling Technology) antibodies. Membranes were then incubated in secondary antibodies (ZB2301; ZSGB-BIO) for 1 h at room temperature. Antibody signals were developed using Immobilon Western chemiluminescent substrate (Millipore, cat. no. WBKLS0100, 200 μl), and the intensities were analyzed using Image J software.

### Statistical analysis

Data are expressed as means ± standard error of the means (SEMs). Paired *t*-tests were used to compare firing rates before and after drug treatment. Data were analyzed using repeated-measures analyses of variance where appropriate. In all cases, *P* < 0.05 was considered significant.

## Results

### Feeding responses after injection of nesfatin-1 into the LPBN

**Figure 1:**
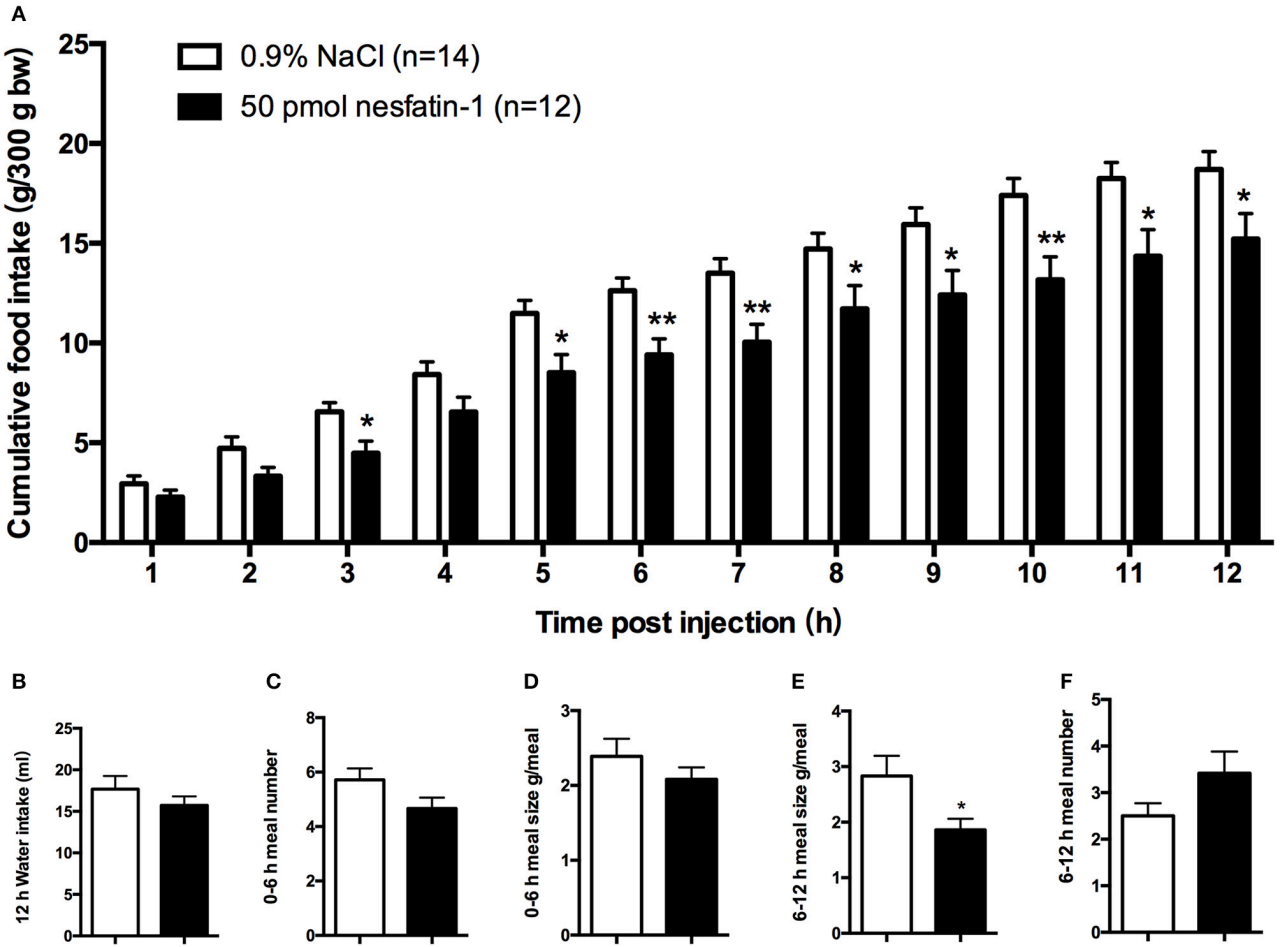
**Feeding responses to injections of nesfatin-1 in the LPBN**. Nesfatin-1 injections (50 pmol) into the LPBN decreased cumulative food intake from 3 to 12 h after injection **(A)** without affecting 12-h water intake **(B)**. Nesfatin-1 (50 pmol/0.5 μl) or vehicle was microinjected into the LPBN of *ad libitum*-fed mice at the onset of the dark phase, and meal numbers at 0–6 h **(C)**, meal sizes at 0–6 h **(D)**, meal sizes at 6-12 h **(E)**, and meal numbers at 6–12 h **(F)** were assessed using an automated food intake-monitoring device. Bars represent the mean ± SEM of 12–14 rats/group. ^*^P < 0.05, ^**^P < 0.01 vs. saline vehicle-injected controls.

We found that, compared with vehicle-treated counterparts, intra-LPBN microinjections of nesfatin-1 (50 pmol) significantly reduced nocturnal cumulative food intake beginning at 5 h (11.49 ± 0.7 vs. 8.52 ± 0.9 g, *P* < 0.05; Figure 1A), which was sustained through 12 h (18.70 ± 0.9 vs. 15.22 ± 1.3 g, *P* < 0.05; Figure 1A). Moreover, intra-LPBN microinjections of nesfatin-1 (50 pmol) had no effect on 12-h water intake (17.47 ± 1.6 vs. 15.72 ± 1.1 ml, *P* > 0.05; Figure 1B).

An analysis of feeding behaviors showed that during the first 6 h, nesfatin-1 had no effect on average meal number or meal size (Figures 1C,D). However, between 6 and 12 h postinjection, compared with those of rats receiving the vehicle, nesfatin-1 decreased the average meal sizes (2.83 ± 0.4 vs. 1.86 ± 0.7 g/meal, *P* < 0.05; Figure 1E) without affecting the meal numbers (Figure 1F).

### Effect of nesfatin-1 in the LPBN on long-term body weight gain

**Figure 2:**
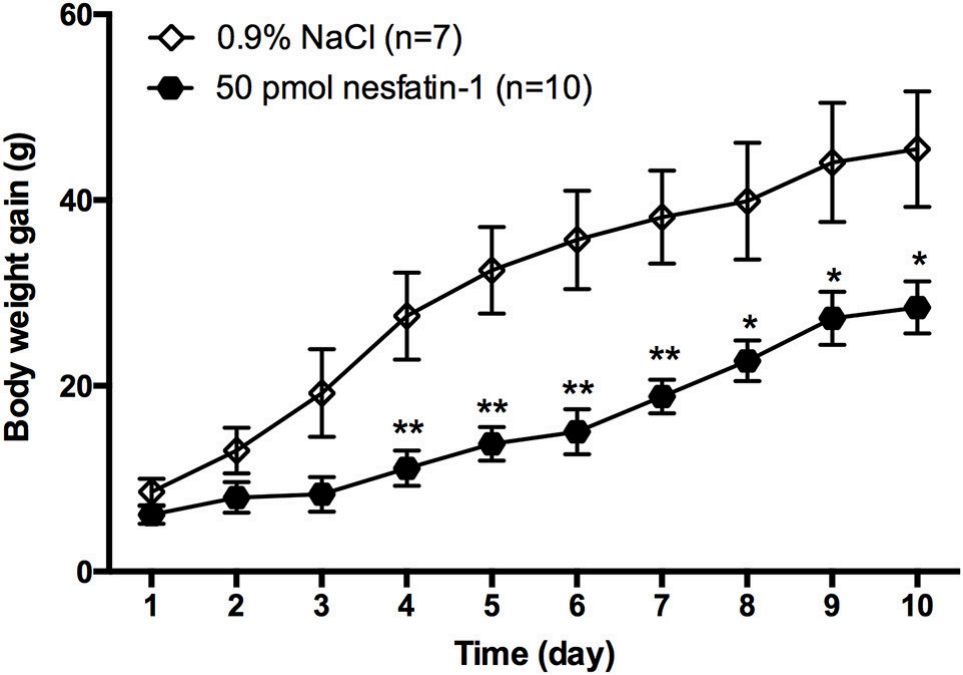
**Change in body weight gained after injections of nesfatin-1 for 10 days**. Amount of body weight gained (increment from day 0) in rats after nesfatin-1 injections (50 pmol/0.5 μl daily). Data are mean ± SEM. ^*^P < 0.05, ^**^P < 0.01 vs. saline vehicle-injected controls.

To determine whether chronic administration of nesfatin-1 causes any changes in long-term body weight gain, rats received microinjections of either nesfatin-1 or saline into the LPBN once daily for 10 days. Compared with saline, nesfatin-1 reduced the amount of body weight gained beginning on day 4 (Figure 2). In the nesfatin-1-treated group (*n* = 10), the amount of body weight gained was reduced by 59.5, 57.6, 57.8, 50.6, 43.0, 37.3, and 42.0% compared with that in the saline-treated group (*n* = 7) on days 4, 5, 6, 7, 8, 9, and 10, respectively.

### Effect of nesfatin-1 on LPBN glucose sensitive neurons *In vivo*

**Figure 3:**
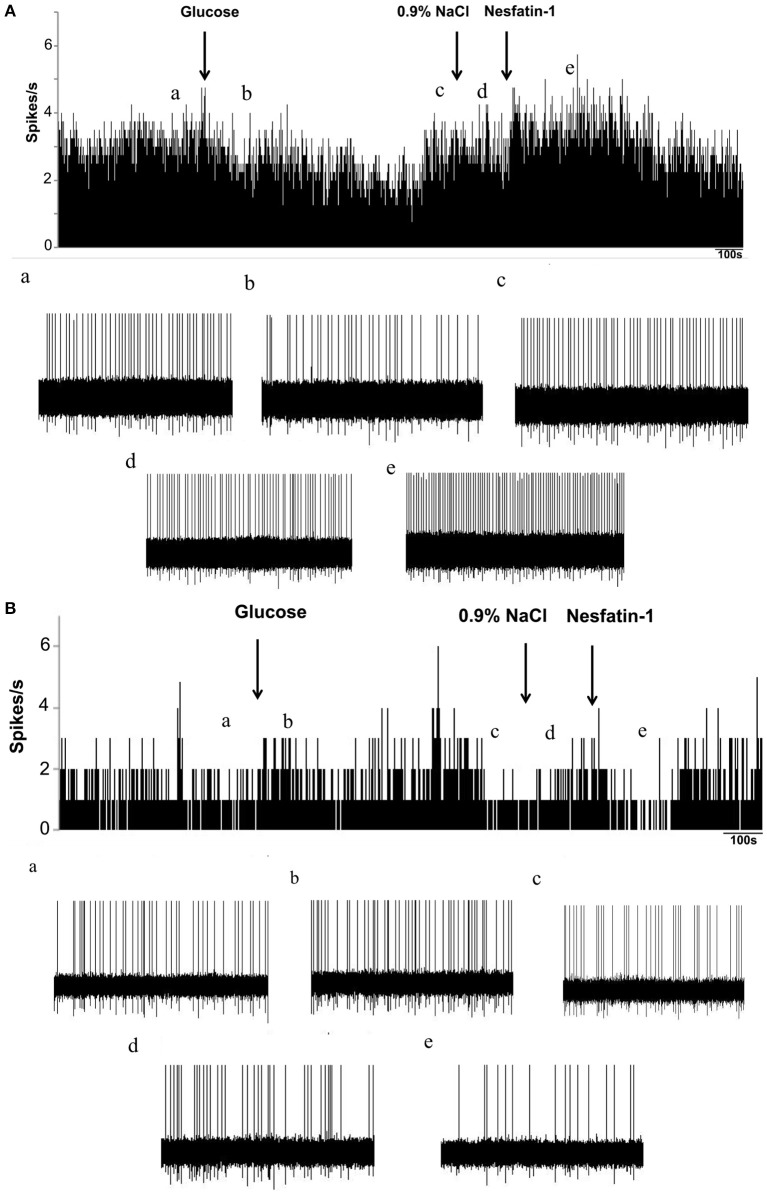
**Effects of nesfatin-1 on glucosensing neurons in the LPBN. (A)** Representative stimulation of a GI neuron in the LPBN by nesaftin-1. The first arrow indicates addition of 5 mM glucose, the second arrow indicates the 0.9% NaCl-treated control, and the third arrow indicates nesfatin-1 (1.5 × 10^−8^ M) application. **(B)** Representative inhibition of a GE neuron by nesaftin-1. **(a)** Baseline before glucose application. **(b)** Firing rate after glucose application. **(c)** Firing rate recovered to baseline before 0.9% NaCl application. **(d)** Firing rate after 0.9% NaCl application and the baseline before nesfatin-1 application. **(e)** Firing rate after nesfatin-1 application.

To investigate whether nesfatin-1 influences spontaneous discharging of LPBN neurons, electrophysiological recordings were made before, during, and after drug injections. Glucose excitatory and inhibitory neurons (GE and GI, respectively) were identified by injecting glucose through the microelectrode. Recordings from 46 neurons in the LPBNs of 44 rats after the application of 5 mM glucose revealed 9 (9/46; 19.6%) that were identified as GE neurons and 24 (24/46; 52.2%) that were identified as GI neurons. Of the 24 GI neurons examined, 14 were activated, 8 were depressed, and 2 failed to respond to nesfatin-1 (Table 1). Injection of nesfatin-1 increased significantly the firing rates of GI neurons from 2.46 ± 0.6 to 4.65 ± 0.8 Hz (*n* = 11, *P* < 0.05; Figure 3A). Of the 9 GE neurons, 3 were activated, 5 were depressed, and 1 failed to respond to nesfatin-1. Nesfatin-1 also decreased significantly the spontaneous firing rates of GE neurons from 3.26 ± 0.4 to 1.67 ± 0.5 Hz (*n* = 4, *P* < 0.05; Figure 3B).

**Table 1:** **Numbers of LPBN neurons responsive to glucose (5 mM) and nesfatin-1 (1.5 × 10^**−8**^)**.

**LPBN neurons (*n* = 47)**	**Nesfatin-1 response**		
	**Inhibited**	**Excited**	**Insensitive**
GI neurons (*n* = 24; 52.2%)	8 (33.3%)	14 (58.3%)	2 (8.3%)
GE neurons (*n* = 9; 19.6%)	5 (55.6%)	3 (33.3%)	1 (11.1%)
Glucose-insensitive neurons (*n* = 14; 30.4%)	3 (21.4%)	0 (0%)	11 (78.6%)

### Influence of SHU9119 on the feeding effects and body weight changes of nesfatin-1

**Figure 4:**
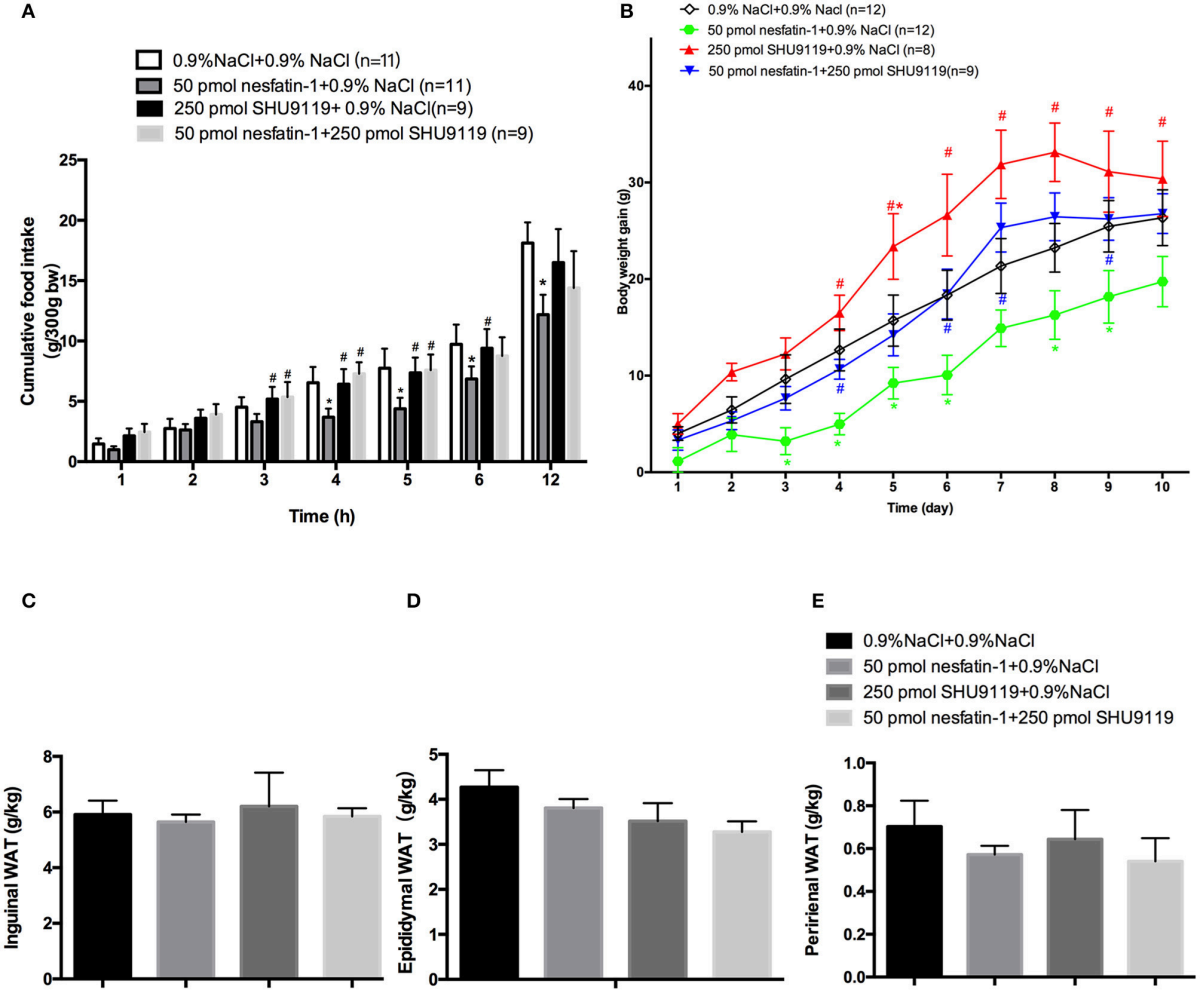
**Effects of SHU9119 on the feeding effects of nesfatin-1. (A)** Effects of SHU9119 on nesfatin-1-induced nocturnal anorexia. Four groups of rats received microinjections (0.5 μl) into the LPBN at the onset of the dark cycle and cumulative food intake was recorded. **(B)** Effects of SHU9119 on nesfatin-1-induced body weight changes. Nesfatin-1-treated animals gained less weight than the other three groups. Rats were sacrificed after 10 days of central injections. **(C–E)** Unilateral WAT from epididymal, inguinal, and perirenal regions was weighed. Data represent the mean ± SEM of 9-11 rats/group. ^*^P < 0.05 vs. saline vehicle-injected controls; ^#^*P* < 0.05 vs. nesfatin-1 group.

**Figure 5:**
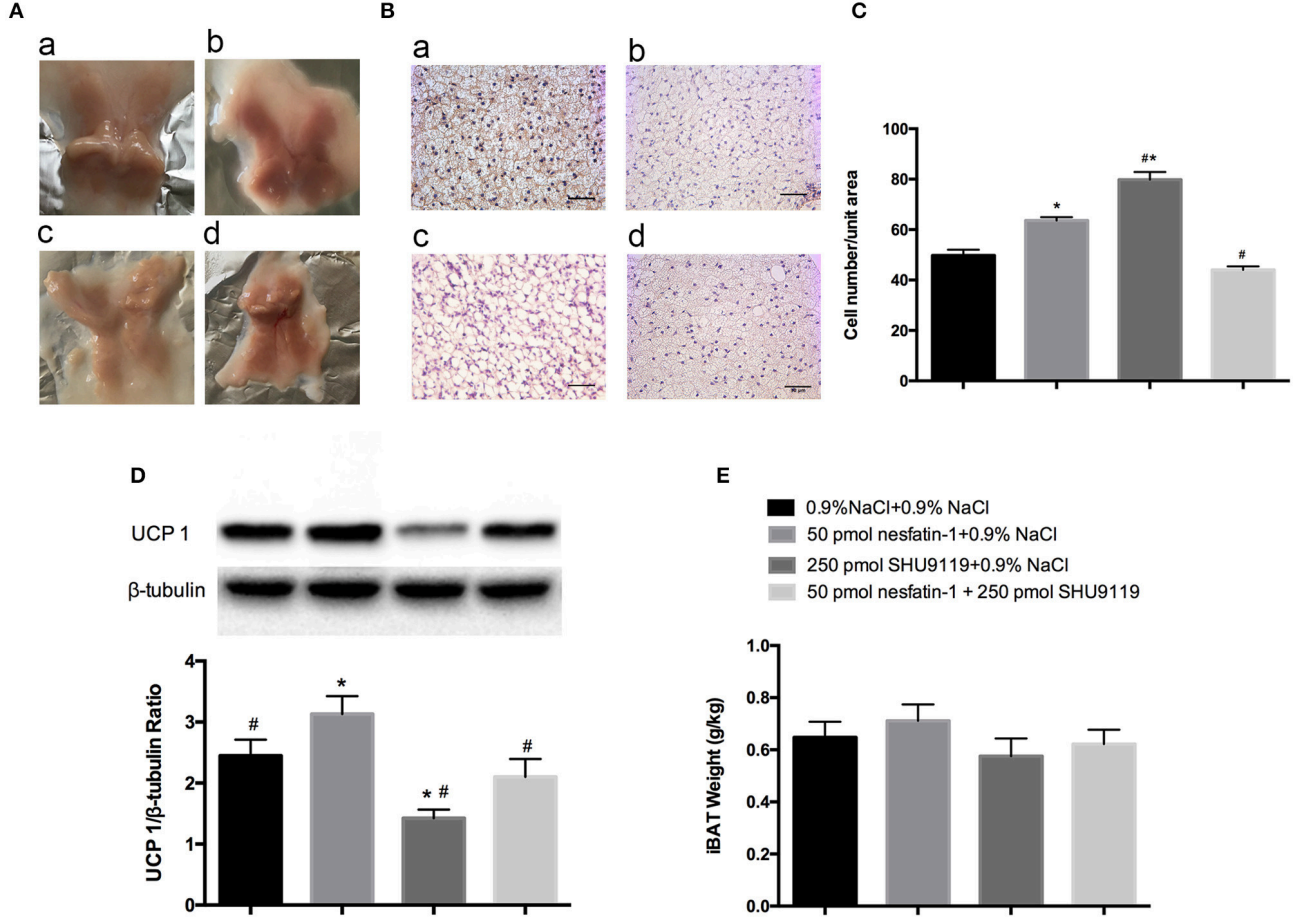
**BAT morphology and UCP1 protein analysis after 10 days of injections into the LPBN**. Fresh interscapular BAT gross samples **(A)** and hematoxylin and eosin staining (200×) **(B)** after 10 days of microinjections into the LPBN; **(a)** 0.9% NaCl group, **(b)** 50 pmol nesfatin-1 group, **(c)** 250 pmol SHU9119 group, **(d)** 50 pmol nesfaint-1 + 250 pmol SHU9119 group. **(C)** Numbers of cell from the same unit areas were counted and recorded. UCP1 protein expression levels in **(D)** and weights of **(E)** BAT were measured. Data represent the mean ± SEM of 7–12 rats/group. ^*^P < 0.05 vs. saline vehicle-injected controls; ^#^*P* < 0.05 vs. nesfatin-1 group.

The administration of SHU9119 abolished the inhibitory effect of nesfatin-1 on cumulative food intake at 4 h (7.28 ± 0.9 g vs. 3.66 ± 0.7 g, *P* < 0.05; Figure 4A) and 5 h (7.57 ± 1.3 g vs. 4.36 ± 0.9 g, *P* < 0.05; Figure 4A). Moreover, SHU9119 attenuated the effect of nesfatin-1 on the amount of body weight gained from day 4 to day 9 (day 4: 5.11 ± 1.2 vs. 10.67 ± 1.2 g, *P* < 0.05; day 9: 16.3 ± 2.5 vs. 25.3 ± 2.5, *P* < 0.05; Figure 4B). When compared with that in the saline-treated group, administration of SHU9119 significantly increased the amount of body weight gained only on day 5 (15.6 ± 2.6 vs. 23.3 ± 3.4 g, *P* < 0.05; Figure 4B).

We next investigated the effects of nesfatin-1 in the LPBN on adipose weight and UCP1 expression. To explore whether nesfatin-1 influences body weight by altering WAT distribution, we weighed inguinal, epididymal, and perirenal fat pads (Figures 4C–E). In addition, the expression of UCP1 in perirenal, epididymal and inguinal WAT was detected by Western blotting. The results show that there were no significant differences between the groups.

### Effect of nesfatin-1 in the LPBN and involvement of the melanocortin system on increasing brown adipose cell numbers and UCP1 protein levels in bat

After 10 days of microinjecting nesfatin-1 into the LPBN, BAT samples appeared darker and redder (Figure 5A) and contained more cells (63.6 ± 1.3 vs. 49.7 ± 2.3 cells/unit area, *P* < 0.05; Figures 5B,C) than vehicle-treated animals. Long-term central injections of nesfatin-1 also increased the protein expression of UCP1 compared with than in vehicle-treated controls (2.39 ± 0.3 vs. 1.67 ± 0.3 AU, *P* < 0.05; Figure 5D).

However, administration of SHU9119 increased the number of lipid droplets and cells (79.8 ± 1.3 vs. 49.7 ± 2.3 cells/unit area, *P* < 0.05; Figures 5B,C) and decreased the expression levels of UCP1 (0.6 ± 0.1 vs. 1.67 ± 0.3 AU, *P* < 0.05; Figure 5D) in BAT compared with those in the vehicle-treated animals, as well as attenuated the nesfatin-1 effects compared with the group receiving nesfatin-1 only. Moreover, no significant differences were observed in the weights of BAT adipose pads (Figure 5E).

## Discussion

Although the role of nesfatin-1 in the hypothalamic nuclei and hindbrain in regulating food intake has been demonstrated (Oh et al., 2006), few studies have investigated the role of nesfatin-1 in the pontine nuclei. Recently, NUCB2/nesfatin-1 immunoreactivity was observed in the LPBN (Goebel et al., 2009, 2011a). Previous studies showed that administration of NUCB2/nesfatin-1 in the third ventricle or at the hindbrain level inhibits nocturnal feeding in rats (Oh et al., 2006; Stengel et al., 2009a). We extend these findings and show that nesfatin-1 in the LPBN induces anorexia between 5 and 12 h after administration, which is similar to that observed with injections in the forebrain. Nesfatin-1 also reduced nocturnal food intake by reducing meal sizes without affecting meal numbers, consistent with the results from a previous report (Goebel et al., 2011b).

Glucose sensitive neurons are located throughout the brain and integrate a variety of hormonal, metabolic, transmitter, and peptide signals involved in regulating energy homeostasis and other biological functions. Studies have shown that glucose sensitive neurons in the hypothalamus and brainstem are involved in glucose homeostasis and glucoprivic feeding (Ritter et al., 1998; Bonnet et al., 2013). There is also increasing interest in glucose sensitive neurons for their role in appetite regulation (Watts and Donovan, 2010). We previously investigated glucose sensitive neurons in hypothalamic nuclei (Chen et al., 2012) and the dorsal vagal complex (Dong et al., 2014) as a possible mechanism for the effects of nesfatin-1 on feeding behavior. As GLP-1 was reported to decrease food intake by increasing the firing rates of LPBN neurons (Richard et al., 2014), we investigated whether nesfatin-1 had a similar effect on a subpopulation of LPBN neurons that were found recently to be part of a glucose sensitive brain region (de Araujo, 2014). Garfield et al. demonstrated that LPBN cholecystokinin neurons are counterregulatory GI neurons whose activity does not impact the first 3 h of food intake (Garfield et al., 2014). In this study, we identified the majority of glucose sensitive neurons in the LPBN as GI neurons, of which 58.3% were excited by nesfatin-1. Thus, nesfatin-1 recapitulates the effect of glucose deprivation on GI neurons and inhibits feeding too. In addition, Wu et al. demonstrated that loss of GABAergic inhibition from agouti-related peptide-producing neurons leads to abnormal activation of the parabrachial nucleus, which in turn inhibits feeding (Wu et al., 2012). Therefore, the mechanism for nesfatin-1's anorexigenic effect is still not completely clear. Further investigation is needed to clarify the final pathways and neurotransmitters impacting the glucose sensitive neurons in the LPBN.

In the central nervous system, nesfatin-1 is thought to be involved both in glucose sensing and in the control of glucose metabolism (Dore et al., 2017). Surprisingly, the areas in the LPBN that are immunoreactive for NUCB2/nesfatin-1 (Goebel et al., 2009, 2011a) and 2-deoxyglucose or insulin-induced c-fos (Garfield et al., 2014) partially overlap. Bonnet et al. (2013) showed that insulin-activated neurons express nesfatin-1 in the hypothalamus and the dorsal vagal complex, supporting the hypothesis that glucoprivation stimulates nesfatinergic neurons. However, studies from Su et al. and Li et al. showed no effect of central nesfatin-1 on blood glucose, glucose tolerance, or insulin sensitivity (Su et al., 2009; Li et al., 2013). Nevertheless, most GI neurons in the LPBN are also nesfatin-1 responsive. Anatomical studies have shown that the LPBN contains at least seven separate subnuclei that can be distinguished by morphology, spatial clustering, and afferent and efferent connectivities (Hayward and Felder, 1999). However, our electrophysiological studies were not restricted to a specific subarea within the LPBN. Further investigation is still needed to determine whether nesfatin-1 functions as a paracrine or autocrine hormone. The identification of a nesfatin-1 receptor(s), as well as subsequent structural-functional analyses, would certainly aid in defining the precise physiological roles of nesfatin-1.

We detected a delayed onset of anorexia after fasting that was induced by nesfatin-1, which corresponds with electrophysiological data (excited GI neurons and inhibited GS neurons) from the PVN (Chen et al., 2012) and the LPBN. The anorexia-related actions of nesfatin-1 result from oxytocin and melatonin signaling in several hypothalamic and medullary anorexigenic pathways (Oh et al., 2006; Maejima et al., 2009; Stengel et al., 2009b; Yosten and Samson, 2009). The LPBN, PVN, and solitary tract nucleus contain oxytocin and pro-opiomelanocortin neurons. Satiety signals from the gut ascend via the afferent vagal nerve to the solitary tract nucleus and continue to the forebrain directly and also indirectly via a relay in the LPBN (Herbert et al., 1990; Karimnamazi et al., 2002; Schwartz, 2009; Grill and Hayes, 2012). Melanocortin-4 receptor-expressing neurons in the PVN project to the LPBN (Garfield et al., 2015), and cholecystokinin-expressing GI neurons in the LPBN target steroidogenic-factor 1-expressing neurons in the ventromedial nucleus of the hypothalamus (Alhadeff et al., 2014a). Although we still do not know the details regarding the neuronal circuitry of the effects of nesfatin-1 in the LPBN, it is possible that nesfatin-1 in the parabrachial nucleus works cooperatively with the PVN pathway.

By administering SHU9119 (250 pmol), we confirmed that nesfatin-1 is involved in the melatonin system, as was found with intracerebroventricular and third ventricle injections in previous studies (Oh et al., 2006; Yosten and Samson, 2009). The evidence also suggests that the anorexigenic action of nesfatin-1 is activated by a hypothalamic-pontine oxytocin-pro-opiomelanocortin-α melanocyte-stimulating hormone-melanocortin 3/4 receptor-signaling pathway (Stengel et al., 2013). In addition to controlling feeding, hypothalamic and hindbrain melanocortin receptors also contribute to energy expenditure (Skibicka and Grill, 2009). The thermogenic actions of nesfatin-1 were first demonstrated by increased core body temperatures after intracerebroventricular administration of nesfatin-1 (25 pmol) (Konczol et al., 2012). Moreover, Wernecke et al. (2014) demonstrated that nesfatin-1 reduces food intake and increases energy expenditure. A possible mechanism of nesfatin-1-induced thermogenesis most likely depends on sympathetic nervous system activation of BAT. The sympathetic nervous system utilizes melanocortin to signal to BAT from the LPBN (Skibicka and Grill, 2009). Nesfatin-1 can also activate the sympathetic nervous system (Yosten and Samson, 2009) and colocalizes with pro-opiomelanocortin (Brailoiu et al., 2007), which suggests that nesfatin-1 might be involved in melanocortin-regulated energy expenditure. In this study, we detected an elevated level of UCP1 in BAT after long-term nesfatin-1 administration, which was abolished by SHU9119. Although we did not measure BAT temperatures, the elevated level of UCP1 protein supports our hypothesis. Nevertheless, nesfatin-1 appears to act as an important negative regulator of energy balance.

A change in body weight can result from altering the balance of food intake and energy expenditure. In fact, we also measured food intake discontinuously during the long-term study after injection of nesfatin-1. On the 3rd day, nesfatin-1's inhibitory effect on food intake was similar to that of the 1st day (as shown in the Supplementary Figure 3). On the 5th day, nesfatin-1 only inhibited cumulative food intake in 6 h (Supplementary Figure 4). On the 7th day, the inhibitory effect of nesfatin-1 on food intake even disappeared (Supplementary Figure 5). Moreover, the body weight gain also showed no difference on the same day (as shown in Figure 4B), which indicated the suppression of food intake might account mainly for the reduction of body weight gain before the 7th day. The food intake inhibiting effect of nesfatin-1 seemed to be lost after 1 week of treatment, maybe due to the compensatory effect of other feeding related peptides. However, the body weight gain still kept reductive tendency till the 10th day, which suggested that the nesfatin-1 thermogenic effect might be responsible for the lower body weight gain during the last few days of our long-term treatment experiment. Compared with nesfatin-1's effect on food intake, its participation in regulating energy expenditure is less well investigated. Intracerebroventricular injections of nesfatin-1 reduce the duration of nocturnal food intake for 2 days and raise body core temperatures (Konczol et al., 2012) and significantly stimulate renal sympathetic nerve activity (Tanida and Mori, 2011), which also acts as an outflow of energy expenditure. Our previous study also showed that nesfatin-1 stimulated free fatty acid utilization in skeletal muscle in T2DM mice (Dong et al., 2013). In the present study, activation of BAT was observed as one of the energy output pathways. Therefore, both inhibition of food intake and promotion of energy expenditure contributed to the reduction of body weight gain after injection of nesfatin-1 in LPBN.

## Conclusion

Nesfatin-1 in the LPBN is involved in energy homeostasis by inhibiting food intake, modulating the excitability of glucose sensitive neurons, and acting on the melanocortin system to enhance UCP1 expression in BAT.

## Author contributions

Conceived and designed the experiments: JD, ZJ, JY, and XC. Performed the experiments: JY, XC, KS, GW, XH, YZ, and DZ. Analyzed the data: JY and XC. Wrote the paper: JY and XC. Edited the manuscript: JD, XC, JY, ZJ, and PC. All authors read and approved the final manuscript.

### Conflict of interest statement

The authors declare that the research was conducted in the absence of any commercial or financial relationships that could be construed as a potential conflict of interest.

## Abbreviations

LPBN: lateral parabrachial nucleus
NUCB2: nucleobindin-2
PVN: paraventricular nucleus
GLP-1: glucagon-like peptide-1
UCP1: uncoupling protein 1
BAT: brown adipose tissue
WAT: white adipose tissue
GE: glucose excitatory
GI: glucose inhibitory.
