# Supplementary figures and images for: Nesfatin-1 in the Lateral Parabrachial Nucleus Inhibits Food Intake, Modulates Excitability of Glucosensing Neurons, and Enhances UCP1 Expression in Brown Adipose Tissue
Source: Front Physiol. 2017 Apr 24;8:235. doi: 10.3389/fphys.2017.00235 (PMC5401881; doi:10.3389/fphys.2017.00235)

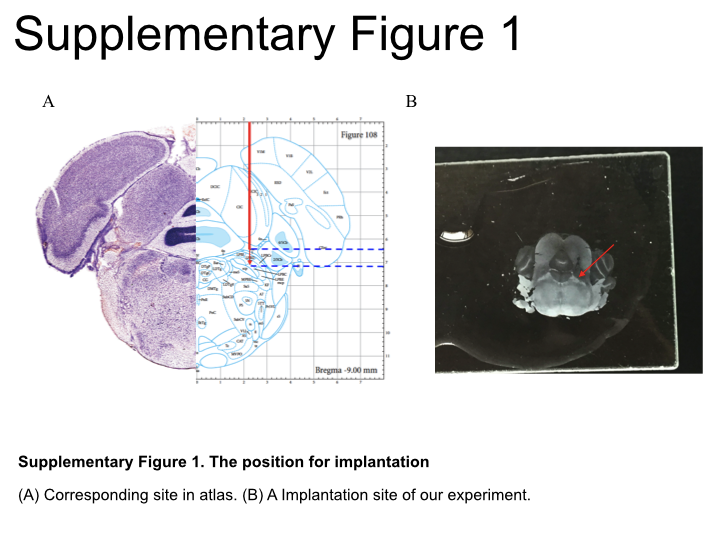

Supplement: Supplementary file 1 [file Image1.TIFF]

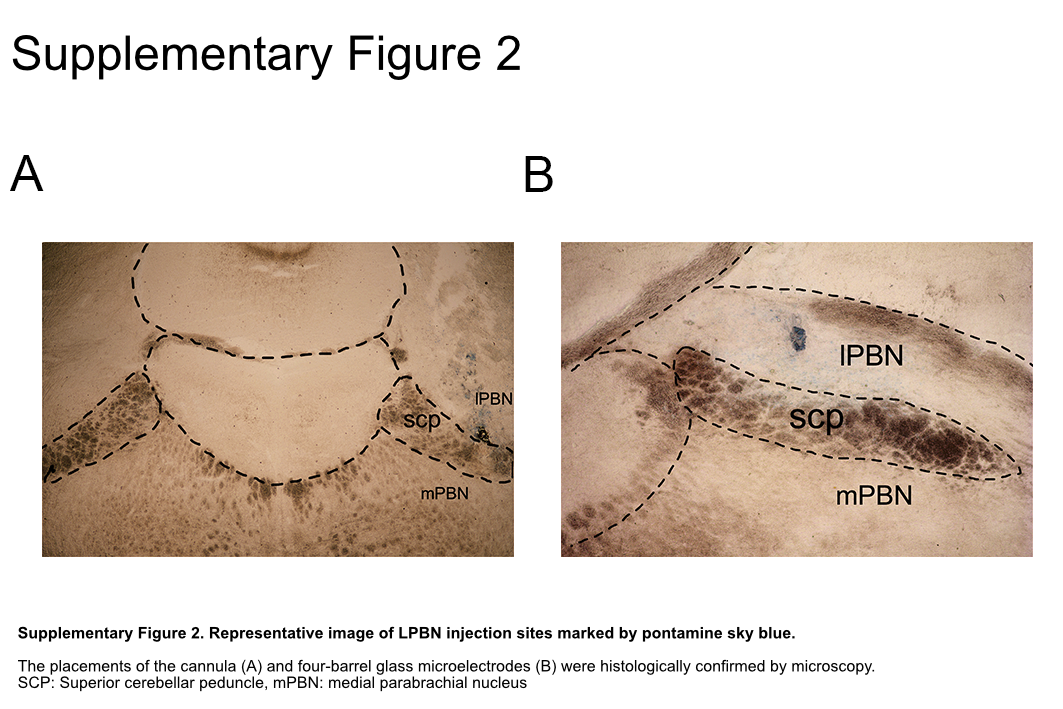

Supplement: Supplementary file 2 [file Image2.TIFF]

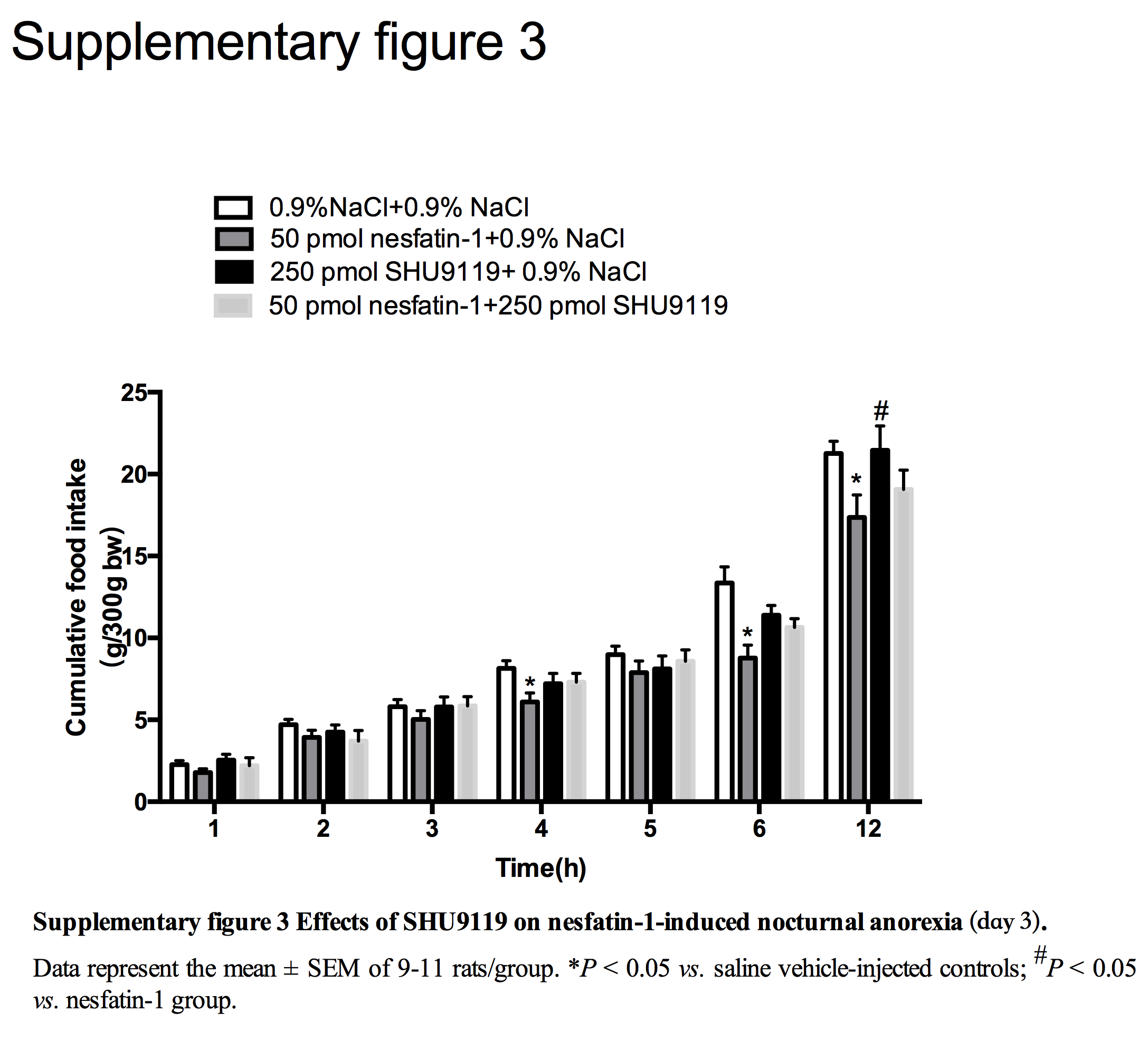

Supplement: Supplementary file 3 [file Image3.TIFF]

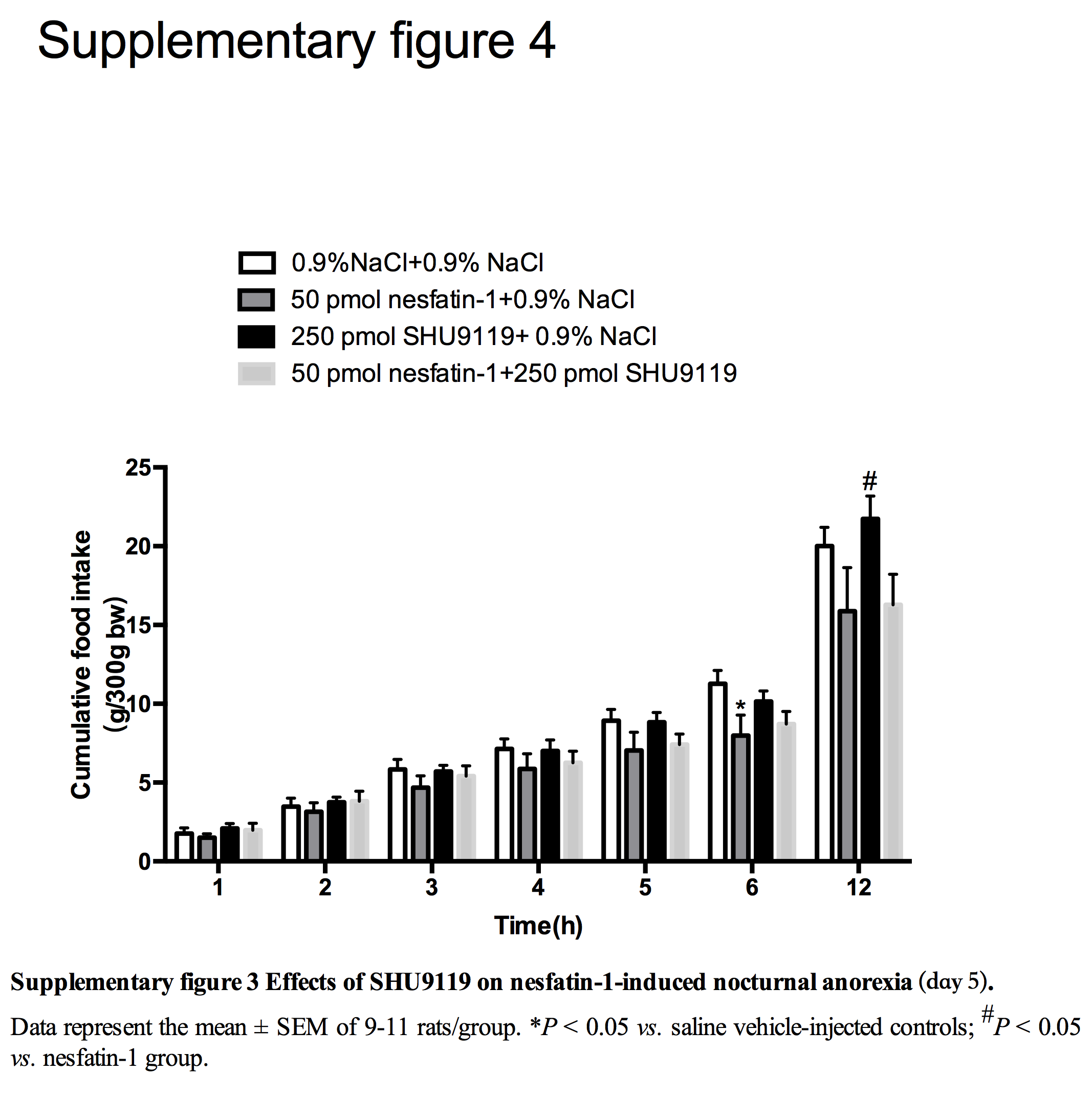

Supplement: Supplementary file 4 [file Image4.TIFF]

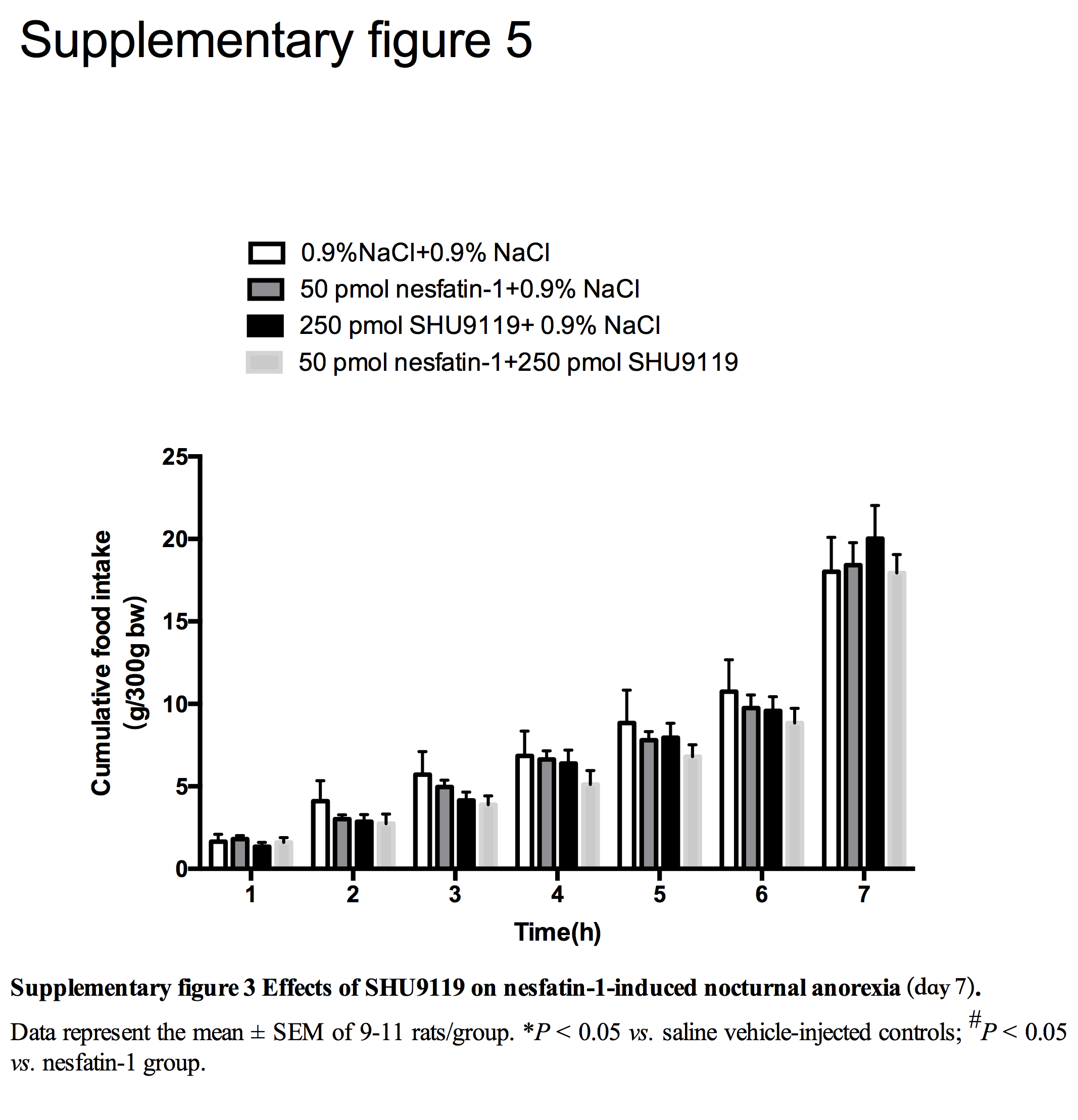

Supplement: Supplementary file 5 [file Image5.TIFF]
